# Understanding the Role of Active Lattice Oxygen in CO Oxidation Catalyzed by Copper-Doped Mn_2_O_3_@MnO_2_

**DOI:** 10.3390/molecules30040865

**Published:** 2025-02-13

**Authors:** Hao Zhang, Tan Meng, Min Zhang, Pengyi Zhang, Peizhe Sun, Huinan Li, Yangyang Yu

**Affiliations:** 1 School of Mechanical Engineering, Tianjin Renai College, Tianjin 301636, China; 15130248376@163.com (H.Z.); tju_zm@126.com (M.Z.); 2 State Key Joint Laboratory of Environment Simulation and Pollution Control, School of Environment, Tsinghua University, Beijing 100084, China; m18612362876@163.com; 3 School of Environmental Science and Engineering, Tianjin University, Tianjin 300072, China; tanmeng@tju.edu.cn (T.M.); sunpeizhe@tju.edu.cn (P.S.)

**Keywords:** CO oxidation, interface region, Cu doping, reactive oxygen species

## Abstract

Although the hopcalite catalyst, primarily composed of manganese oxide and copper oxide, has been extensively studied for carbon monoxide (CO) elimination, there remains significant potential to optimize its structure and activity. Herein, Cu-doped Mn_3_O_2_@MnO_2_ catalysts featuring highly exposed interfacial regions were prepared. The correlation between interfacial exposure and catalytic activity indicates that the interfacial region serves as the active site for CO catalytic oxidation. The characteristic adsorption of CO by Cu species significantly enhances the catalytic activity of the catalyst. And XPS and ICP-OES analyses reveal that Cu ions coexist in both the interlayer and lattice of δ-MnO_2_. Furthermore, XPS analysis was employed to quantify the average oxidation state (AOS) of Mn and the molar ratios of oxygen species, demonstrating that both surface-adsorbed oxygen and surface lattice oxygen act as reactive oxygen species in the catalytic reaction, playing a crucial role in CO oxidation. Notably, the surface reactive oxygen species influence the adsorption of CO onto Cu species, and the replenishment of these reactive species is identified as the rate-limiting step in the CO catalytic oxidation process.

## 1. Introduction

Carbon monoxide (CO), which has a strong affinity for hemoglobin, is one kind of toxic gas for humans [1]. The Air Quality Guidelines (AQGs) issued by the WHO in 2021 state that the indoor AQG level for CO should not exceed 4 mg/m^3^. Prolonged exposure to CO can lead to severe health issues, including neurological damage and cardiovascular diseases. Effectively removing CO is crucial for mitigating these risks, ensuring a safer environment, and improving public health. There are various technologies available for the removal of CO, and among them, the low-temperature catalytic removal of CO stands out as a particularly simple and efficient method. This approach not only effectively reduces CO levels, but also operates under milder temperature conditions, making it a practical choice for various applications.

In the past few decades, significant efforts have been dedicated to the development of high-performance catalytic materials. Noble metal catalysts, such as gold, platinum, silver, and palladium, have garnered extensive attention due to their exceptional catalytic properties and moisture resistance [2,3,4,5,6]. Researchers have made considerable strides in understanding single atom and nanoparticle catalysis, leading to advancements in various applications. However, the high cost of these noble metals, coupled with the stringent conditions required for their preparation, pose substantial challenges for their practical use in everyday applications. As a result, the design and development of non-noble metal catalysts with excellent performance have become a central focus for researchers in the field. Among various non-noble metal catalysts, hopcalite catalyst (manganese oxide and copper oxide as the main components) has been widely explored to eliminate CO and show remarkable catalytic performance [7,8,9,10]. Hopcalite catalysts are currently widely used as commercial catalysts for catalytic CO removal. However, they also have some limitations, including poor catalytic activity and deactivation upon exposure to moisture. Despite their effectiveness in CO oxidation under certain conditions, their performance can degrade significantly when exposed to water or under less-than-ideal operating environments [8]. This has led to ongoing research aimed at improving the stability and efficiency of hopcalite catalysts, focusing on enhancing their resistance to moisture and increasing their overall catalytic activity for their broader application in environmental and industrial processes.

Although the hopcalite catalyst demonstrates potential for CO oxidation, its performance remains constrained by an unclear structure–activity relationship. Research indicates that several factors, such as crystal phase, the ratio of cations with different valence states, and the exposed surface facets, can have a substantial impact on the performance of catalysts [11,12,13]. However, the structure–activity relationship of these catalysts remains inadequately understood, highlighting a crucial area for further investigation. Moreover, it has been observed that the reactive oxygen species generated by different catalysts can vary significantly depending on the target pollutants being addressed [14]. Therefore, synthesizing catalysts with high active regions and abundant reactive oxygen species is not only a promising avenue for improving catalytic efficiency, but also represents a meaningful contribution to the advancement of environmental remediation technologies.

The mismatch in lattice constants between different materials often leads to the formation of numerous defects at the interface of the heterojunction. These defects, such as dislocations, vacancies, and interstitials, can play a significant role in modulating the electronic properties of the materials involved [15,16,17,18,19,20]. In catalytic applications, the presence of these defects can provide additional active sites that enhance the reactivity of the catalyst, improving its overall performance. In addition, these defects can also enrich active oxygen species and increase the material’s surface area, all of which are critical factors in enhancing catalytic activity. In recent years, researchers have synthesized catalysts with heterojunction structures and investigated the catalytic activity of the heterojunction interface [21,22]. Moreover, recent studies have shown that CO oxidation activity is closely linked to the presence of Cu^+^ carbonyl species [23,24,25,26,27], and that the characteristic adsorption of CO by Cu^+^ enhances the catalytic activity of the catalyst. Additionally, the synthesis of highly dispersed Cu species has been recognized as an effective approach to improving catalyst performance [28,29].

In this study, heterogeneous catalysts with highly exposed interfacial regions were synthesized through a redox reaction between KMnO_4_ and Mn_2_O_3_, and catalysts with different Cu content were obtained by adding Cu(NO_3_)_2_ into the reaction system. Scanning electron microscopy (SEM) was employed to analyze the morphology and interfacial exposure of the catalysts, allowing us to establish a relationship between interfacial exposure and catalytic activity. Based on the correlation between the content of surface-adsorbed oxygen (as determined by X-ray photoelectron spectroscopy, XPS) and catalytic activity, along with the interactions of surface-adsorbed oxygen and surface lattice oxygen with CO (assessed through temperature-programmed desorption, CO-TPD), this study provides a detailed discussion of the reactive oxygen species involved in the CO oxidation process.

## 2. Results and Discussion

### 2.1. Crystal Structure and Morphology

Upon heating to high temperatures in the presence of air, MnCO_3_ reacts with O_2_ to produce Mn_2_O_3_, releasing CO_2_ (Equation (1)). Then, MnO_2_ is grown over Mn_2_O_3_ through a redox reaction between Mn_2_O_3_ and KMnO_4_ under acidic conditions (Equation (2)). When copper nitrate is added to the reaction solution, Cu ions are doped into the MnO_2_ as the redox reaction goes on.

(1) $$MnCO_{3}+O_{2}\to MnO_{2}+CO_{2}$$

(2) $$Mn_{2}O_{3}+H^{+}+MnO_{4}^{-}\to MnO_{2}+H_{2}O$$

The catalysts are labeled numerically (e.g., 0.04, 0.2, 0.7, 1Cu, 10Cu, and 50Cu) based on the molar ratios of key precursors used in their synthesis. A detailed description of the naming convention is provided in Section 3.2.2 and Section 3.2.3.

**Figure 1:**
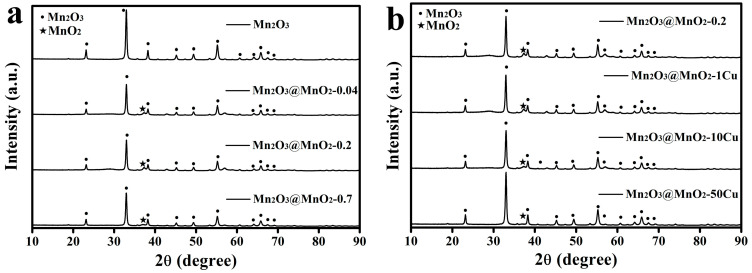
(**a**) XRD patterns of Mn_2_O_3_ and series of Mn_2_O_3_@MnO_2_ catalysts. (**b**) XRD patterns of Mn_2_O_3_@MnO_2_-0.2 and series of Cu-doped Mn_2_O_3_@MnO_2_ catalysts.

To investigate the phase structures of the synthesized Mn_2_O_3_ and MnO_2_, XRD patterns of the catalysts are tested and shown in Figure 1. The diffraction peaks marked with circles match well with JCPDS 41-1442 (bixbyite). The diffraction peak marked with star (2θ = 37.3°) is well indexed to (−111) of δ-MnO_2_ according to the standard card PDF# 80-1098. Characteristic peaks of Mn_2_O_3_ are presented in all XRD patterns of the samples, indicating an excess of Mn_2_O_3_ in the redox reaction between Mn_2_O_3_ and KMnO_4_. Notably, the (−111) characteristic peak for Mn_2_O_3_@MnO_2_-0.7 is weaker than that for Mn_2_O_3_@MnO_2_-0.2 (Figure 1a), suggesting a significant decrease in the crystallization quality of δ-MnO_2_ with increasing amounts of KMnO_4_ in the reaction system. Furthermore, the (−111) characteristic peak of the samples decreases when increasing the mass of Cu(NO_3_)_2_·3H_2_O in the reaction system, indicating that Cu doping adversely affects the crystallinity of δ-MnO_2_.

**Figure 2:**
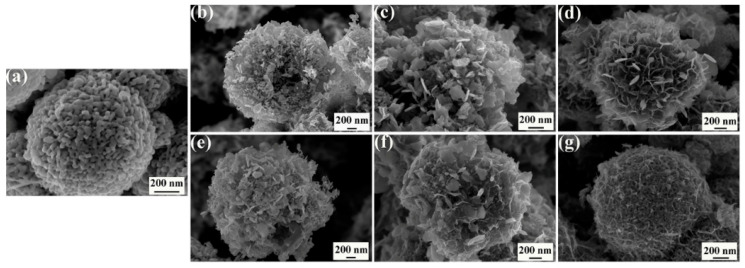
SEM images of prepared samples: (**a**) Mn_2_O_3_, (**b**) Mn_2_O_3_@MnO_2_-0.04, (**c**) Mn_2_O_3_@MnO_2_-0.2, (**d**) Mn_2_O_3_@MnO_2_-0.7, (**e**) Mn_2_O_3_@MnO_2_-1Cu, (**f**) Mn_2_O_3_@MnO_2_-10Cu, and (**g**) Mn_2_O_3_@MnO_2_-50Cu.

Figure 2 shows the SEM images of the prepared samples. As shown in Figure 2a, Mn_2_O_3_ exhibits a porous spherical morphology, which serves as an important structural foundation for the subsequent growth of δ-MnO_2_. In Figure 2b,c, it can be observed that the δ-MnO_2_ nanosheets are oriented almost perpendicular to the surface of Mn_2_O_3_, with their morphology varying significantly depending on the extent of the redox reaction, while the mesoporous spherical structure of Mn_2_O_3_ remains distinctly observable. When the KMnO_4_ increases to a certain extent in the reaction solution, the δ-MnO_2_ nanosheets adopt a flower-like morphology (Figure 2d). This alteration in morphology severely affects the exposure of Mn_2_O_3_, and markedly reduces the accessibility of the interface between Mn_2_O_3_ and δ-MnO_2_. In addition, the incorporation of Cu ions has a significant impact on the morphology of δ-MnO_2_. As shown in Figure 2e,f, the δ-MnO_2_ nanosheets become thinner with the incorporation of Cu ions. And when the Cu(NO_3_)_2_·3H_2_O increases to a certain extent in the reaction solution, most of the δ-MnO_2_ nanosheets fall down (Figure 2g), and it also seriously influences the exposure of the interface between Mn_2_O_3_ and δ-MnO_2_.

**Figure 3:**
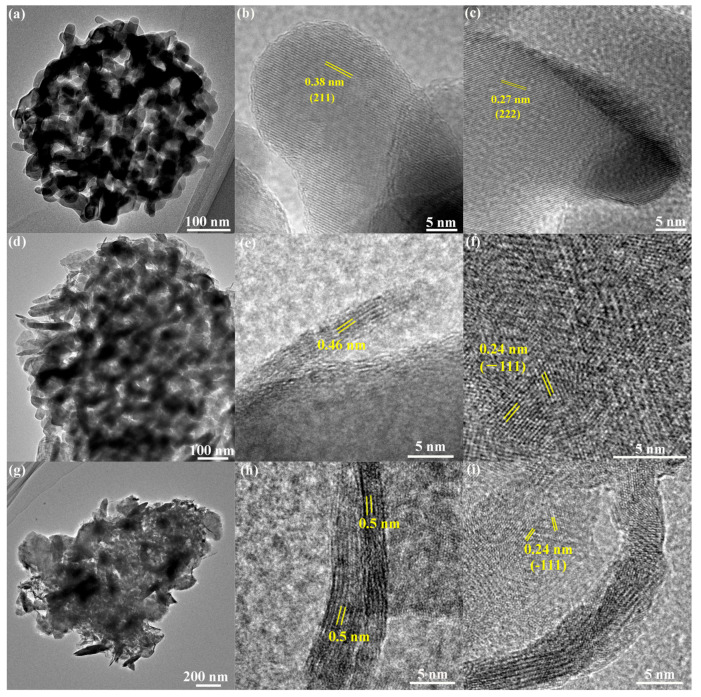
TEM and HRTEM images of Mn_2_O_3_ (**a**–**c**); Mn_2_O_3_@MnO_2_-0.2 (**d**–**f**); and Mn_2_O_3_@MnO_2_-10Cu (**g**–**i**).

HRTEM images were taken to further investigate the lattice parameters of δ-MnO_2_, as depicted in Figure 3. The regularly arranged lattice fringes of 0.38 nm and 0.27 nm in Figure 3b,c correspond to the interplanar distances of the (211) and (222) facets of Mn_2_O_3_, respectively. The crystalline quality of δ-MnO_2_ is poor, while the lattice fringes of 0.24 nm corresponding to the interplanar distances of (−111) can be vaguely observed. This observation is consistent with the findings from XRD analysis. Moreover, δ-MnO_2_ has a lamellar structure formed by [MnO_6_] octahedral, and the interbedded cations and water molecules maintain the stability of the layered structure. As shown in Figure 3e,h, the interplanar distances of (001) facet of δ-MnO_2_ are 0.46 nm and 0.5 nm, which are smaller than previous reports [30,31,32,33]. Figure S1 displays the TEM images of the Mn_2_O_3_@MnO_2_-10Cu sample before and after 350 °C treatment, and it proves that the smaller interplanar distance of (001) facet of δ-MnO_2_ is caused by heat treatment. The following factor may be responsible for the above phenomenon: a large amount of interlayer water was removed (as illustrated in Figure S2), and the collapse of the interlayer structure occurred in the roasting process, resulting in the reduction in layer spacing.

### 2.2. Catalytic Activity for CO Oxidation

**Figure 4:**
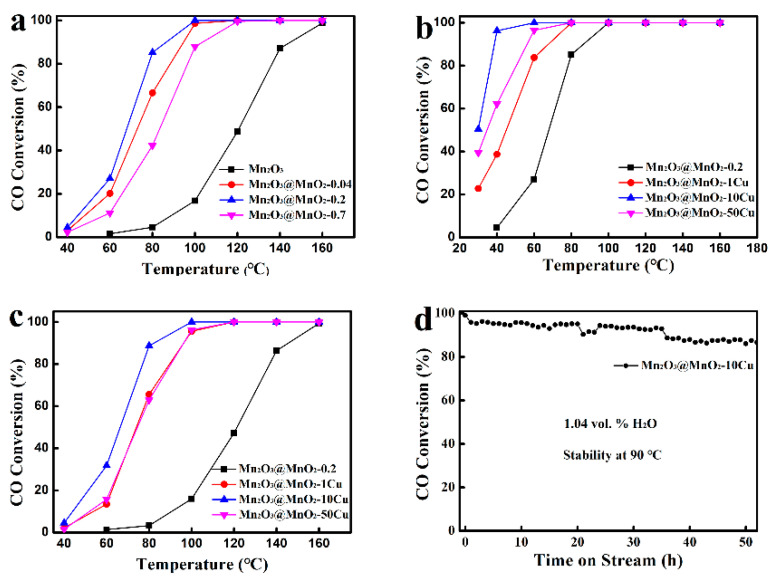
(**a**,**b**) CO conversion as functions of reaction temperature over different catalysts (test condition: dry gas), (**c**) CO conversion as functions of reaction temperature over different catalysts (test condition: in the presence of 1.04 vol.% H_2_O), and (**d**) stability test for Mn_2_O_3_@MnO_2_-10Cu (test condition: in the presence of 1.04 vol.% H_2_O).

The catalytic performance of prepared samples for CO catalytic oxidation was investigated, as shown in Figure 4. By increasing the amount of δ-MnO_2_ formation over Mn_2_O_3_, the Mn_2_O_3_@MnO_2_ catalysts exhibit “volcanic” characteristics (Figure 4a). Notably, Mn_2_O_3_@MnO_2_-0.2 exhibits better catalytic performance than Mn_2_O_3_ and Mn_2_O_3_@MnO_2_-0.7, which is fully covered with δ-MnO_2_. This suggests that neither δ-MnO_2_ nor Mn_2_O_3_ serves as the primary active species in CO catalytic oxidation. The correlation between the exposure of the interface region and the catalytic activity should be noted, and the interface region is considered to be the active region for CO catalytic oxidation. Additionally, the influence of Cu doping on the performance of Mn_2_O_3_@MnO_2_ was studied. As the Cu content in the Mn_2_O_3_@MnO_2_ catalyst increased, the performance of the catalyst also exhibited “volcanic” characteristics (Figure 4b). Specifically, the introduction of a small amount of Cu^2+^ into the catalyst resulted in a decrease in the complete conversion temperature of CO, indicating that Cu^2+^ enhances the catalytic oxidation of CO. However, excessive Cu doping adversely affected the morphology of δ-MnO_2_ (as shown in Figure 2g) and the exposure of the interface region, leading to a decline in catalytic performance. This further corroborates the notion that the interface region is the active site for CO catalytic oxidation.

Up to now, it has always been a challenge to enhance the moisture resistance and durability of the catalyst. Based on this, we investigated the moisture resistance of the catalyst in the presence of 1.04 vol.% H_2_O. Although the catalysts exhibited reduced catalytic activity compared to dry conditions, they still maintained catalytic performance (Figure 4c). As illustrated in Figure S3, Δ*T*_100_ of Mn_2_O_3_@MnO_2_-10Cu is 40 °C, and that of Mn_2_O_3_@MnO_2_-0.2 is about 60 °C (*T*_100_ denotes the temperature at which the CO conversion reached 100%, and the difference between *T*_100_ under dry and wet conditions is expressed as Δ*T*_100_). This indicates that the incorporation of Cu^2+^ improves the moisture resistance of the catalyst to a certain extent. As shown in Figure 4d, there is a decrease in the CO conversion rate over 50 h of testing, but it can still reach 88% at the end of our testing, indicating that the Mn_2_O_3_@MnO_2_-10Cu catalyst has excellent water resistance under this moisture level.

### 2.3. Physicochemical Properties of As-Prepared Catalysts

**Figure 5:**
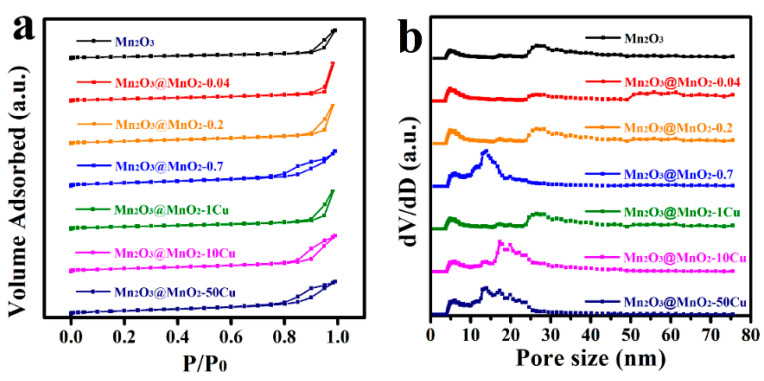
(**a**) Nitrogen adsorption–desorption curves and (**b**) BJH pore distributions of catalysts.

The nitrogen adsorption–desorption curves and BJH pore distributions are depicted in Figure 5. As illustrated in Figure 5a, the N_2_ adsorption—desorption isotherms for all catalysts exhibit a type IV classification, characterized by hysteresis loops indicative of capillary condensation occurring in the mesopores present on the catalyst surfaces. Furthermore, the presence of a type H_3_ hysteresis loop suggests the existence of slit-shaped pores within the catalysts. In addition, the content and morphology of δ-MnO_2_ are critical factors affecting the *S*_BET_, and the growth of δ-MnO_2_ nanosheets over Mn_2_O_3_ increased the *S*_BET_ of the catalysts (Table 1). According to Figure 5b, the pore size distribution of Mn_2_O_3_ is primarily mesoporous (2–50 nm), with predominant pore sizes of approximately 4.9 nm to 26.4 nm. The formation of δ-MnO_2_ significantly reduced the size of the larger mesopores (approximately 26.4 nm), resulting in a decrease to 13.9 nm, while still remaining within the mesoporous range. Furthermore, the incorporation of Cu adversely affected the crystallinity and structural integrity of the δ-MnO_2_ nanosheets, resulting in the collapse of the nanosheets and the blockage of the mesopores (e.g., approximately 26.4 nm).

**Figure 6:**
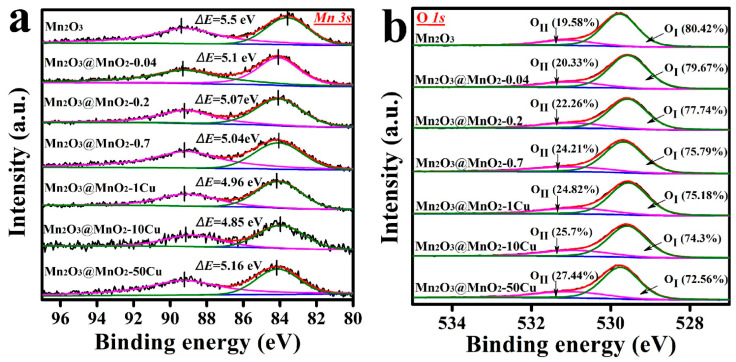
XPS spectra of (**a**) Mn 3s and (**b**) O 1s in prepared samples: Mn_2_O_3_, Mn_2_O_3_@MnO_2_-0.04, Mn_2_O_3_@MnO_2_-0.2, Mn_2_O_3_@MnO_2_-0.7, Mn_2_O_3_@MnO_2_-1Cu, Mn_2_O_3_@MnO_2_-10Cu, and Mn_2_O_3_@MnO_2_-50Cu.

XPS analysis was conducted to explore the average oxidation state (AOS) of Mn and the molar ratios of oxygen species. The Mn 3s spectra and the AOS of Mn, calculated using Equation (3), are shown in Figure 6a and Table 1. As anticipated, the AOS of Mn increased with the formation of MnO_2_, following the trend: Mn_2_O_3_ (2.763) < Mn_2_O_3_@MnO_2_-0.04 (3.219) < Mn_2_O_3_@MnO_2_-0.2 (3.247) < Mn_2_O_3_@MnO_2_-0.7 (3.281). Notably, the AOS of Mn displayed “volcanic” characteristics with increasing Cu content in the MnO_2_@Mn_2_O_3_ catalyst, and this may be related to the doping location of Cu ions. One possibility is that Cu ions could incorporate into the octahedra of δ-MnO_2_ by substituting Mn. According to the principle of electric neutrality, the replacement of high valence Mn^4+^ by low valence Cu^2+^ would lead to an increase in the AOS of Mn. It can be seen from Table 1 that the AOS of Mn in Mn_2_O_3_@MnO_2_-1Cu and Mn_2_O_3_@MnO_2_-10Cu are higher than that in Mn_2_O_3_@MnO_2_-0.2, implying that Cu has incorporated into the octahedra of δ-MnO_2_ by substituting Mn. In addition, it is widely reported that K^+^ exists in the interlayer of δ-MnO_2_ for charge balance [34,35]. Based on the ICP-OES results, an increase in Cu content corresponds with a decrease in K content in the Cu-doped catalysts, indicating that Cu likely substitutes for the original K^+^ ions in the interlayer of δ-MnO_2_. To further verify the presence of Cu ions in the mezzanine of δ−MnO_2_, Cu-doped catalysts were dispersed in 100 mL KNO_3_ solution and stirred at 60 °C for 6 h. After treatment with KNO_3_ solution, Cu content decreased and K content increased in the catalysts (Table S1), indicating a distinct ion replacement procedure between Cu ions and K ions. The substitutability of Cu ions also confirms that Cu ions exist in the interlayer of δ−MnO_2_. The replacement of low valence K^+^ by high valence Cu^2+^ in the mezzanine of δ-MnO_2_ provides more positive charges. According to the principle of electric neutrality, the replacement of low valence K^+^ by high valence Cu^2+^ will decrease the AOS of Mn, and this has also been verified experimentally. As shown in Figure S4, the replacement of high valence Cu^2+^ by low valence K^+^ increases the AOS of Mn. The Mn_2_O_3_@MnO_2_-50Cu possesses a lower AOS than other Cu-doped samples, and this may be attributed to the high concentration of Cu^2+^ in the interlayer of the δ−MnO_2_. Based on the above results and discussion, we can conclude that Cu is doped into the interlayer and lattice of δ-MnO_2_ simultaneously.

(3) AOS=8.956−1.126×ΔE

where Δ*E* represents the binding energy difference between characteristic peaks of Mn 3s spectra.

Figure 6b shows the O 1s spectra of different samples. All the O 1s spectra were deconvoluted into two distinct peaks, with binding energies located at ~529.6 eV and ~531.0 eV, corresponding to lattice oxygen (O_I_) and surface adsorbed oxygen (O_II_), respectively. As the formation of δ-MnO_2_ over Mn_2_O_3_ increased, the ratio of O_II_/O_I_ increased from 0.24 to 0.32. In addition, the ratio of O_II_/O_I_ increased from 0.29 to 0.38 with an increase in the Cu content in Mn_2_O_3_@MnO_2_ catalysts. These results indicated that the formation of δ-MnO_2_ and Cu doping were favorable to the adsorption of oxygen species on the surface of the catalyst. Oxygen vacancies, which serve as critical active sites for many oxidation reactions, are the primary sites for oxygen species adsorption on the catalyst surface [36]. A higher ratio of O_II_/O_I_ indicates an increased formation of oxygen vacancies. The adsorbed oxygen species are widely recognized as reactive oxygen species in the catalytic oxidation process [37,38,39]. Notably, our study indicates that there is no direct linear correlation between the amount of adsorbed oxygen species and the catalytic activity of the synthesized catalysts. This suggests that adsorbed oxygen may not be the sole species influencing catalytic performance. Consequently, further investigation into the reducibility of surface lattice oxygen and the lattice oxygen storage capacity of the prepared catalysts is warranted.

**Figure 7:**
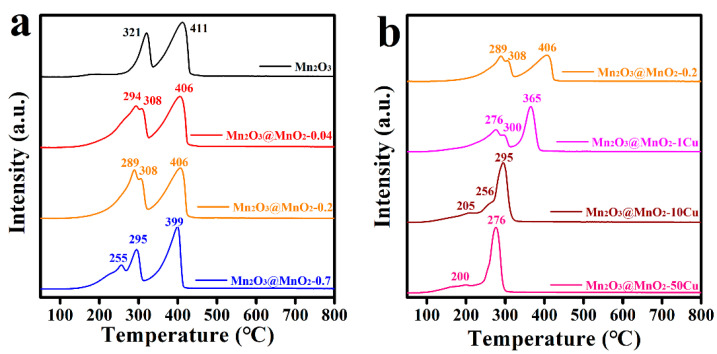
(**a**) H_2_-TPR profiles of prepared Mn_2_O_3_@MnO_2_ samples with different amount of MnO_2_, (**b**) H_2_-TPR profiles of prepared Mn_2_O_3_@MnO_2_-0.2 with varying levels of Cu doping.

To understand the reducibility of the prepared catalysts, the H_2_-TPR profiles were measured and collected. As shown in Figure 7, Mn_2_O_3_ exhibited two distinct reduction peaks at 321 °C and 411 °C, corresponding to the conversion processes of Mn_2_O_3_ → Mn_3_O_4_ → MnO. With the formation of δ-MnO_2_, pre-peaks (294 °C, 289 °C, 255 °C) emerged, which were attributed to the reduction of MnO_2_ to Mn_2_O_3_. It is well known that a lower initial reduction temperature indicates a stronger migration ability of lattice oxygen [40,41]. Notably, the initial reduction temperature of the catalysts decreased from 294 °C to 255 °C with the increase in δ-MnO_2_ formation. Furthermore, the initial reduction temperature of the catalysts was further reduced by Cu doping. Intriguingly, there was also no correlation between the catalytic activity and mobility of lattice oxygen. Consequently, the oxygen storage capacity of the prepared catalysts was investigated, and the O_2_-TPD profiles are shown in Figure S5. The O_2_ desorption signals were identified as physically and chemically adsorbed oxygen species (desorption temperature below 300 °C), surface lattice oxygen (desorption temperature 300–600 °C), and bulk lattice oxygen (desorption temperature above 600 °C) [41,42]. With the increase in δ-MnO_2_ formation and Cu doping content, the amount of surface lattice oxygen decreased. It is an interesting phenomenon that the increase in reducibility is accompanied by the decrease in oxygen storage capacity for the Cu-doped catalysts. Based on these observations, we speculate that the optimal balance between oxygen storage capacity and the mobility of lattice oxygen occurs in Mn_2_O_3_@MnO_2_-10Cu, which possesses the maximum amount of reactive oxygen species available for the oxidation of CO.

**Figure 8:**
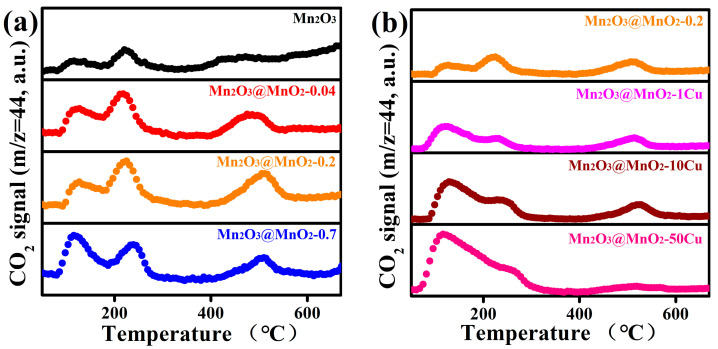
(**a**) CO-TPD-MS profiles of prepared Mn_2_O_3_@MnO_2_ samples with different amount of MnO_2_, (**b**) CO-TPD-MS profiles of prepared Mn_2_O_3_@MnO_2_-0.2 with varying levels of Cu doping.

To verify the above conjecture, CO-TPD-MS was employed to detect CO_2_ (Figure 8), which is the sole product formed during CO oxidation, with the peak areas summarized in Table S2. The CO_2_ desorption between 60 °C and 300 °C was derived from the reaction between CO and the adsorbed oxygen on the catalyst surface. The amount of CO_2_ desorption follows the following sequence: Mn_2_O_3_@MnO_2_-0.04 < Mn_2_O_3_@MnO_2_-0.2 < Mn_2_O_3_@MnO_2_-0.7 < Mn_2_O_3_@MnO_2_-1Cu < Mn_2_O_3_@MnO_2_-10Cu < Mn_2_O_3_@MnO_2_-50Cu. This is consistent with the amount of adsorbed oxygen species on the catalyst surface in the XPS results. Meanwhile, we should note that Mn_2_O_3_@MnO_2_-0.2 and Mn_2_O_3_@MnO_2_-10Cu, which exhibit relatively good catalytic performance, released more CO_2_ than the other samples around 500 °C. The CO_2_ desorption around 500 °C resulted from the reaction between CO and the surface lattice oxygen, which indicates that surface lattice oxygen is an important oxygen species affecting catalyst activity. In summary, both surface-adsorbed oxygen and surface lattice oxygen are essential contributors to the oxidation of CO.

### 2.4. Analysis of Surface Reaction Process

**Figure 9:**
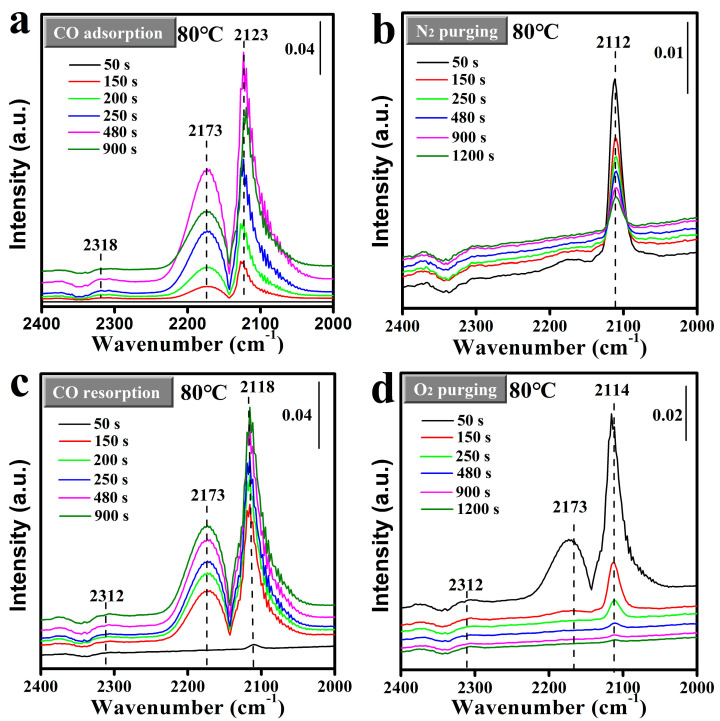
In situ DRIFTS study of (**a**) CO adsorption, (**b**) N_2_ purging, (**c**) CO resorption, and (**d**) O_2_ removal on Mn_2_O_3_@MnO_2_-10Cu. The catalysts were pretreated in situ at 200 °C under mixture gas (50% O_2_ and 50% N_2_) flow in the DRIFTS reaction cell before data collection (5% CO flow rate; 10 mL·min^−1^; N_2_ flow rate; 20 mL·min^−1^; O_2_ flow rate; 20 mL·min^−1^; and temperature, 80 °C).

To gain further insight into the CO adsorption behavior on the surface of the Mn_2_O_3_@MnO_2_-10Cu catalyst, in situ DRIFTS was employed to study the adsorption dynamics of CO in a “CO-N_2_-CO-O_2_” mode at a temperature of 80 °C (as depicted in Figure 9). The first and second adsorption processes of CO on the catalyst surface were marked as CO-adsorption-I and CO-adsorption-II, respectively. Bands in the range of 2105~2120 cm^−1^ were attributed to the CO adsorption on Cu^+^ species [23,43], while the peaks at ~2170 cm^−1^ were attributed to gaseous CO accompanied by CO-Cu^+^ species [43]. Additionally, bands around 2312 cm^−1^ were linked to the presence of gaseous CO_2_ [44]. As illustrated in Figure 9, CO adsorption on Cu^+^ species (Cu^+^-CO) was detected around 2120 cm^−1^, accompanied by the gaseous CO peak at 2173 cm^−1^. However, there is a significant difference in the CO adsorption behaviors in the CO-adsorption-I and CO-adsorption-II processes: in the CO-adsorption-I process, the Cu^+^-CO peak increases first and then decreases as the adsorption time is extended; while the Cu^+^-CO peak in the CO-adsorption-II process shows a trend of continuous increscent with the extending adsorption time. It should be pointed out that the Cu^+^-CO peak intensity reached its maximum at 480 s (~0.19) in the CO-adsorption-I process, and it was higher than that at 900 s (~0.18) in the CO-adsorption-II process. Furthermore, the behavior of CO adsorption on Cu^+^ species at 30 °C was found to be similar to that at 80 °C (shown in Figure S6). Based on the above results, we hypothesize that the adsorbed CO can react with the reactive oxygen species, resulting in the reduction in reactive oxygen species on the surface of the catalyst. And the reactive oxygen species were beneficial to the adsorption of CO on Cu species. Firstly, with the decrease in reactive oxygen species on the surface of the catalyst, the adsorption capacity of Cu^+^ to CO was weakened, and the CO adsorption peak decreased after 480s in the CO-adsorption-I process. Subsequently, the reactive oxygen species on the surface of the catalyst were not replenished during the scavenging process of N_2_, resulting in the adsorption capacity of Cu^+^ to CO in the CO-adsorption-II process being weaker than that in the CO-adsorption-I process. In order to verify the above conjecture, an in situ DRIFTS study of CO adsorption on Mn_2_O_3_@MnO_2_-10Cu at 120 °C was conducted, and is shown in Figure S7. The gaseous CO_2_ appears at 200 s, and the peak intensity increases first and then decreases with the extending adsorption time. Furthermore, the Cu^+^-CO peak also increases first and then decreases with the extending adsorption time. This proves that the adsorbed CO does react with the reactive oxygen species to generate CO_2_, resulting in the reduction in reactive oxygen species on the surface of the catalyst, and the surface reactive oxygen species of the catalyst related to the adsorption of CO on Cu species.

**Figure 10:**
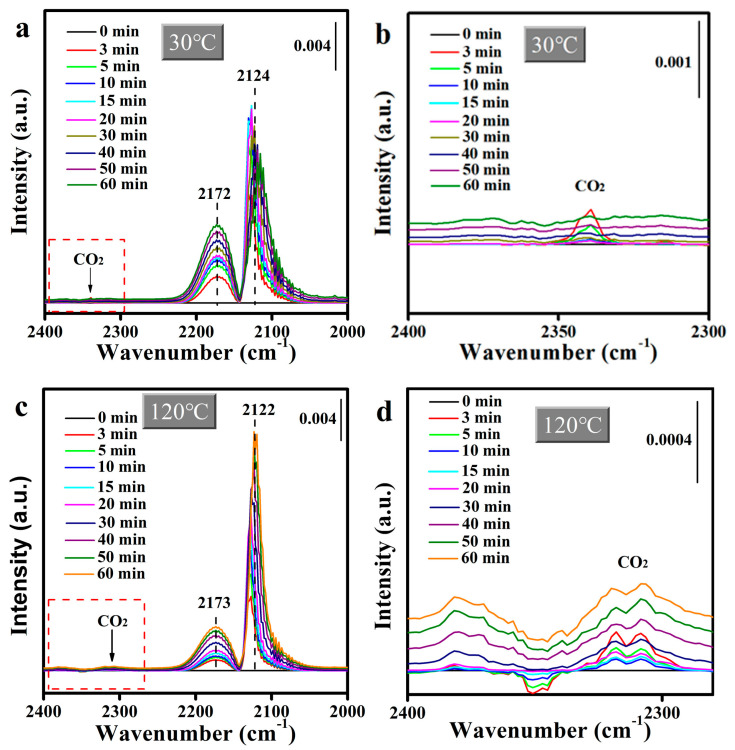
In situ DRIFTS taken for 60 min under the reaction conditions (2% CO/O_2_ flow) at (**a**) 30 °C and (**c**) 120 °C for Mn_2_O_3_@MnO_2_-10Cu. (**b**) and (**d**) are partially enlarged images of (**a**) and (**c**), respectively.

To elucidate the rate-limiting step of CO catalytic oxidation, in situ DRIFTS spectra were recorded under steady-state conditions using a reactant mixture for 60 min. Figure S8 displays the in situ DRIFTs spectra of Mn_2_O_3_@MnO_2_-10Cu exposed to the flow of 120 ppm CO at 110 °C. The band at 2324 cm^−1^ for gaseous CO_2_ was detected, indicating the CO adsorbed on Cu^+^ had been oxidized, while the peak at ~2120 cm^−1^, which was attributed to the CO adsorption on the Cu^+^ sites, was not detected. This demonstrates that the CO adsorbed on Cu^+^ can be rapidly transferred and oxidized by reactive oxygen species on the surface of the catalyst, and that the transfer of CO on Cu^+^ is not the rate-limiting step of CO catalytic oxidation. In addition, in situ DRIFTs spectra of Mn_2_O_3_@MnO_2_-10Cu under the reaction condition (2% CO/O_2_ flow) at 30 °C and 120 °C were recorded and are shown in Figure 10a and Figure 10c, respectively. Figure 10b,d are partial enlargements of Figure 10a and Figure 10c, respectively. The peak intensity for gaseous CO_2_ gradually decreased and eventually almost disappeared at 30 °C, while the peak intensity for gaseous CO_2_ remained basically stable with the reaction time extending at 120 °C. As discussed above, the adsorbed CO can be oxidized by reactive oxygen species on the surface of the catalyst. In Figure 10a,c, the band at ~2124 cm^−1^ for Cu^+^-CO is detected, indicating that CO is sufficient in the catalytic oxidation process. The reaction between CO and reactive oxygen species disappeared gradually at 30 °C, indicating that incoming CO reduces the reactive oxygen species, and that the replenishment of reactive oxygen species is very slow under the current conditions. While CO_2_ is continuously generated at 120 °C, indicating that the reactive oxygen species of the catalyst can be supplemented to a certain extent at 120 °C, from the above, we can conclude that the replenishment of reactive oxygen species is the rate-limiting step of CO catalytic oxidation. There is another important phenomenon necessary to point out. At 30 °C, when the replenishment of reactive oxygen species is slow, the Cu^+^-CO peak increases first and then decreases with the extending reaction time, while at 120 °C, when the reactive oxygen species can be quickly replenished, the Cu^+^-CO peak does not decrease with the extending reaction time. And it once again proves our previous speculation: the surface reactive oxygen species of the catalyst is beneficial to the adsorption of CO on Cu species.

In the presence of water, the catalytic activity of Mn_2_O_3_@MnO_2_-10Cu decreased remarkably. To further investigate this phenomenon, we examined the effect of water on the adsorption of CO on Cu^+^. Figure S9 presents the in situ DRIFTS spectra of Mn_2_O_3_@MnO_2_-10Cu exposed to a flow of 2% CO under conditions of RH = 100% at 25 °C. The Cu^+^-CO peak nearly vanished compared to the spectra obtained in the absence of moisture, indicating that the adsorption capacity of Cu^+^ for CO is significantly diminished due to the presence of water. This attenuation of CO adsorption is a critical factor contributing to the observed reduction in catalyst activity in the presence of water.

## 3. Materials and Methods

### 3.1. Chemicals and Reagents

The information on chemical reagents and manufacturers can be found in the Supporting Materials (Text S1).

### 3.2. Catalyst Preparation

#### 3.2.1. Mn_2_O_3_ Nanosphere

The Mn_2_O_3_ nanosphere was obtained through MnCO_3_ thermal decomposition at 600 °C for 4 h under air.

#### 3.2.2. Mn_2_O_3_@MnO_2_

A series of Mn_2_O_3_@MnO_2_ catalysts was synthesized through a redox reaction between Mn_2_O_3_ and KMnO_4_ under acidic conditions. Initially, 1 g of Mn_2_O_3_ nanospheres was dispersed in 150 mL of deionized water using ultrasonic and magnetic agitation treatment (solution I). A specific amount of KMnO_4_ and 600 μL of HCl were then dissolved in another 150 mL of deionized water (solution II). Solution II was added to solution I, and the resulting mixture was stirred at 90 °C for 30 min. The solid product was then filtered, washed (the catalysts were washed by deionized water until the filtrate was neutral), dried, and calcinated at 350 °C for 5 h in the air. The amounts of KMnO_4_ in solution II were varied, with 0.04 g, 0.2 g, and 0.7 g selected for the synthesis. The resulting catalysts were labeled as Mn_2_O_3_@MnO_2_-0.04, Mn_2_O_3_@MnO_2_-0.2, and Mn_2_O_3_@MnO_2_-0.7, respectively.

#### 3.2.3. Cu-Doped Mn_2_O_3_@MnO_2_

Furthermore, 1 g of Mn_2_O_3_ nanospheres was dispersed in 150 mL of deionized water using ultrasonic and magnetic agitation (solution I). In a separate container, 0.2 g of KMnO_4_, 600 μL of HCl and a specific amount of Cu(NO_3_)_2_·3H_2_O were dissolved in 150 mL of deionized water (solution III). Solution III was then added to solution I, and the resulting mixture was stirred at 90 °C for 30 min. The solid product was filtered, washed, dried, and calcinated at 350 °C for 5 h in the air. The amounts of Cu(NO_3_)_2_·3H_2_O in solution III were varied, with 0.012 g, 0.122 g, and 0.611 g selected for use. The corresponding molar ratios of Cu^2+^ to the generated MnO_2_ were designed to be 1:100, 10:100, and 50:100. And the catalysts were denoted as Mn_2_O_3_@MnO_2_-1Cu, Mn_2_O_3_@MnO_2_-10Cu, and Mn_2_O_3_@MnO_2_-50Cu, respectively.

### 3.3. Materials Characterization and Catalytic Activity Tests

The details of the material characterization equipment and performance testing are provided in the Supporting Materials (Texts S2 and S3).

## 4. Conclusions

In this study, Cu-doped Mn_2_O_3_@MnO_2_ heterojunction catalysts were prepared through a redox reaction between Mn_2_O_3_ and KMnO_4_ for CO elimination. The interfacial region of Mn_2_O_3_ and MnO_2_ demonstrated higher catalytic activity compared to either component alone. The Mn_2_O_3_@MnO_2_-10Cu catalyst achieved remarkable 100% CO conversion at 60 °C, highlighting its potential for practical applications. XPS and ICP-OES analysis indicated that Cu^1+/2+^ ions exist both in the lattice and interlayer of δ-MnO_2_. Furthermore, surface lattice oxygen, alongside surface-adsorbed oxygen, acts as a reactive oxygen species crucial for the catalytic process. Importantly, the replenishment of these reactive species was the rate-limiting step in CO catalytic oxidation. These insights not only deepen our understanding of the catalytic mechanisms in Cu-doped Mn_2_O_3_@MnO_2_ systems, but also underscore their potential for developing efficient catalysts in environmental remediation, particularly for air pollution control.

## Figures and Tables

**Table 1:** Several physicochemical parameters of catalysts.

Sample	Mn (ICP, wt%)	Cu (ICP, wt%)	K (ICP, wt%)	BET (m^2^·g^−1^)	Pore Diameter (nm)	AOS of Mn	XPSO _II_/O_I_
Mn_2_O_3_	73.7	_	_	51.5	4.9/26.4	2.763	0.24
Mn_2_O_3_@MnO_2_-0.04	69.2	_	0.010	59.7	4.9/26.4	3.219	0.26
Mn_2_O_3_@MnO_2_-0.2	69.45	_	0.057	80.2	4.9/26.4	3.247	0.29
Mn_2_O_3_@MnO_2_-0.7	71.51	_	0.34	110.9	4.9/13.9	3.281	0.32
Mn_2_O_3_@MnO_2_-1Cu	70.93	0.29	0.071	67.0	4.9/26.4	3.371	0.33
Mn_2_O_3_@MnO_2_-10Cu	68.03	1.33	0.066	93.0	4.9/17.3	3.495	0.35
Mn_2_O_3_@MnO_2_-50Cu	68.25	2.17	0.025	107.8	4.9/13.9	3.146	0.38

## Data Availability

The experimental data used to support the results of this study are available in the article and in the Supplementary Materials.

## Supplementary Materials

The following supporting information can be downloaded at https://www.mdpi.com/article/10.3390/molecules30040865/s1. Text S1: Chemicals and reagents. Text S2: Materials characterization. Text S3: Catalytic activity tests. Table S1: ICP-OES results of samples. Table S2: The peak area of CO_2_ desorption signal. Figure S1: TEM images of samples. Figure S2: TG curve of Mn_2_O_3_@MnO_2_-10Cu. Figure S3: T_100_ of different catalysts under dry and wet gas conditions. Figure S4: XPS spectra. Figure S5: O_2_-TPD profiles of different samples. Figures S6–S9: In situ DRIFTS study of different samples.
