# Supplementary material for: Understanding the Role of Active Lattice Oxygen in CO Oxidation Catalyzed by Copper-Doped Mn2O3@MnO2
Source: Molecules. 2025 Feb 13;30(4):865. doi: 10.3390/molecules30040865 (PMC11858229; doi:10.3390/molecules30040865)
Supplement: Supplementary file 1 [file molecules-30-00865-s001.zip › molecules-3456392-supplementary.pdf]

Supporting Information

Understanding the role of active lattice oxygen in CO oxidation catalyzed by copper-doped Mn_2O_3@MnO_2

Hao Zhang[1,3#], Tan Meng[2#], Min Zhang[1], Pengyi Zhang[3*], Peizhe Sun[2], Huinan Li[3], Yangyang Yu[1*]

1 School of Mechanical Engineering, Tianjin Renai College, Tianjin, 301636, China

2 School of Environmental Science and Engineering, Tianjin University, Tianjin, 300072, China

3 State Key Joint Laboratory of Environment Simulation and Pollution Control, School of Environment, Tsinghua University, Beijing, 100084, China

* Corresponding author

E-mail address: zpy@tsinghua.edu.cn (Pengyi Zhang)

E-mail address: yuyangyang@tju.edu.cn (Yangyang Yu)

[#] These authors contributed equally to this work.

Text S1 Chemicals and reagents

Manganese carbonate ($MnCO_3$, CP), potassium permanganate ($KMnO_4$, 99.5%) nitric acid (HNO_3, AR 65%-68%) and potassium nitrate (KNO_3, 99 %) were purchased from Sinopharm Chemical Reagent Co. Ltd. Copper nitrate trihydrate ($Cu(NO_3)_2 \cdot 3H_2O$, 99%) was obtained from J&K. Hydrochloric acid (HCl, AR) purchased from Beijing Chemical Works. All the chemicals and reagents were used as received without further purification.

Text S2 Materials characterization

The X-ray diffraction (XRD) patterns were obtained on the Bruker X-ray diffractometer (D8-Advance, Germany) instrument equipped with Cu-Kα X-ray source. SEM images were recorded on a Merlin SEM microscope (Carl Zeiss, Germany). TEM images were acquired on a JEM-2011 transmission electron microscope (JEOL, JEM-2100 Japan) operated at 150 kV. X-ray photoelectron spectroscopy (XPS) were measured on an ESCALAB 250Xi X-ray photoelectron spectrometer (Thermo Fisher, USA). Binding energies were calibrated with the C 1s peak at 284.8 eV. The specific surface area and pore size distribution were determined on a Micromeriticis ASAP 2020 analyzer (USA), using the nitrogen adsorption data at 77 K. The element contents of catalysts were determined by inductively coupled plasma-optical emission spectroscopy (ICP-OES, Thermo IRIS Intrepid II XSP).

H_2 temperature programmed reduction (H_2-TPR), O_2 temperature programmed desorption (O_2-TPD) and CO temperature programmed desorption (CO-TPD) were performed on an AutoChem II 2920 instrument (Micromeritics, USA) equipped with a thermal conductivity detector (TCD). Prior to testing, 50 mg samples (40-60 meshes) were placed in a quartz tube and pretreated at 105 °C for 30 min in the helium flow. For the H_2-TPR measurement, the sample was programmed to rise to 800 °C at a ramp rate of 5 °C/min in 5% H_2/Ar atmosphere. For the O_2-TPD analysis, the sample was treated with 5% O_2/He for 30 min and purged by helium flow for another 30 min. Subsequently, it was heated from 40 °C to 800 °C at a ramp rate of 5 °C/min in the helium flow. For the CO-TPD measurement, the sample was treated with 5% CO/He for 30 min and

purged by helium flow for another 30 min, then the sample was was heated from 50 °C to 800 °C at a ramp rate of 5 °C/min in the helium flow.

In-situ diffuse reflectance infrared Fourier transform spectra (DRIFTS) was performed on a Nicolet 6700 FTIR (Thermo Fisher, USA) equipped with an in-situ cell to detect the change of sample surface during CO oxidation. Before testing, the sample was pretreated at 200 ℃ for 120 min in nitrogen flow. After the sample was cooled down to 30 ℃, 140 ppm CO was injected into the in-situ cell at a flow rate of 200 $mL·min^{-1}$ with the synthetic air as the balance gas.

In order to further understand the adsorption behavior of CO on the catalyst surface, the gas supply of the in-situ cell follows the following process: CO-N_2-CO-O_2. Prior to starting the in situ DRIFTS measurement, the sample was pretreated at 200 ℃ for 120 min under flowing gas mixture (50% O_2 and 50% N_2). After the sample was decreased to designed temperature, gas mixture (5% CO and He balance) was injected into the in-situ cell at a flow rate of 10 $mL·min^{-1}$. Then, pure N_2, CO and pure O_2 are individually fed into the in-situ cell. The IR spectra were collected at different purging time.

Text S3 Catalytic activity tests

The CO catalytic performance of the catalyst was evaluated in a fixed-bed quartz tube reactor. 100 mg samples (40-60 mesh) were placed in a quartz tube with the inner diameter of 6 mm. The quartz tube reactor was placed in a pipe furnace with programmable temperature control. The reaction temperature was designed and each reaction temperature was maintained for seventy minutes. 140 ppm CO balanced with synthesized air was continuously injected into the reactor, and the total flow rate of was 200 $mL·min^{-1}$ with the corresponding gas hourly space velocity (GHSV) of 120 $L·g^{-1}·h^{-1}$. The CO conversion ratio was calculated using the following equation:

$$\text{CO conversion} = \frac{C_{inlet} - C_{outlet}}{C_{inlet}} \times 100\% \qquad (1)$$

where C_{inlet} and C_{outlet} are the molar concentration of CO in the inlet and outlet, respectively.

Table S1. ICP-OES results of as-prepared and KNO_3 solution treated samples

Sample	Mn (ICP, wt%)	Cu (ICP, wt%)	K (ICP, wt%)	Cu/K mol ratio
Mn_2O_3@MnO_2-10Cu	70.1	1.18_	0.075	9.66
KNO_3 solution treated Mn_2O_3@MnO_2-10Cu	70.4	0.623	0.311	1.23
Mn_2O_3@MnO_2-50Cu	58.7	2.11	0.051	24.92
KNO_3 solution treated Mn_2O_3@MnO_2-50Cu	61.5	1.49	0.564	1.62

Table S2. The peak area of CO_2 desorption signal

Sample	Peak area between 60°C and 300°C (E-08)	Peak area between 400°C and 600°C (E-08)
Mn_2O_3@MnO_2-0.04	6.42	2.52
Mn_2O_3@MnO_2-0.2	6.52	**3.45**
Mn_2O_3@MnO_2-0.7	6.87	2.32
Mn_2O_3@MnO_2-1Cu	9.66	3.01
Mn_2O_3@MnO_2-10Cu	16.8	**4.06**
Mn_2O_3@MnO_2-50Cu	28.1	1.22

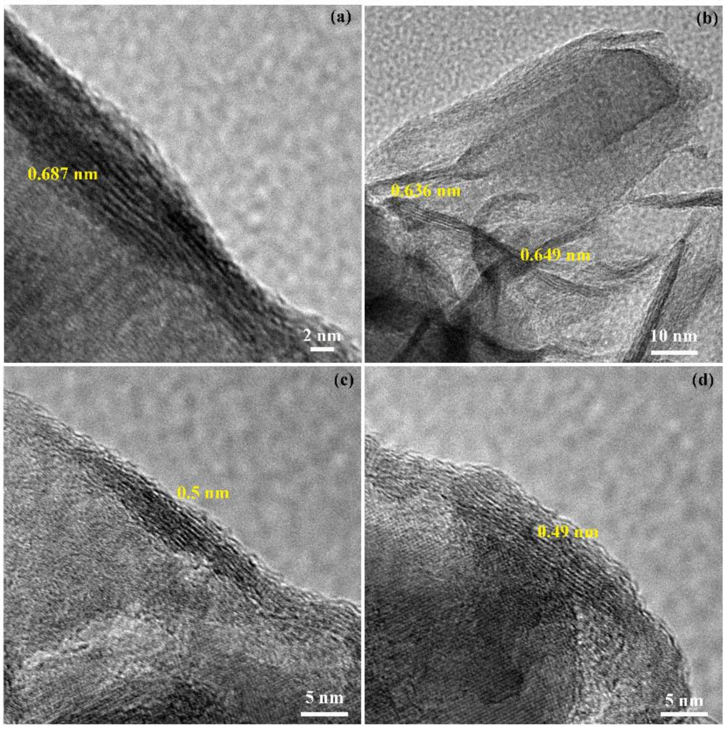

Figure S1. (a) and (b) the TEM images of Mn_2O_3@MnO_2-10Cu sample before 350℃ treatment, (c) and (d) the TEM images of Mn_2O_3@MnO_2-10Cu sample after 350℃ treatment.

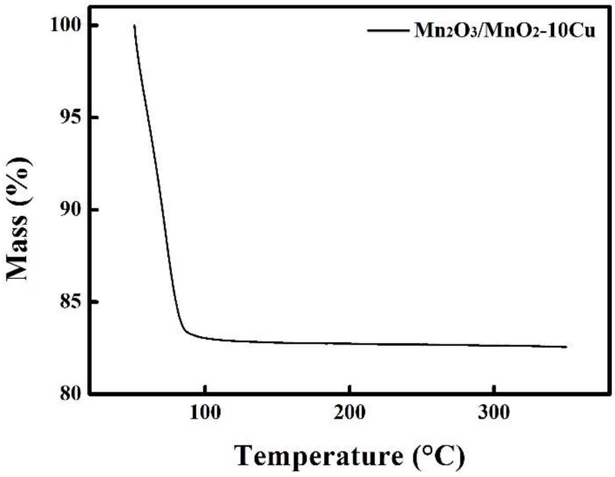

Figure S2. TG curve of Mn_2O_3@MnO_2-10Cu.

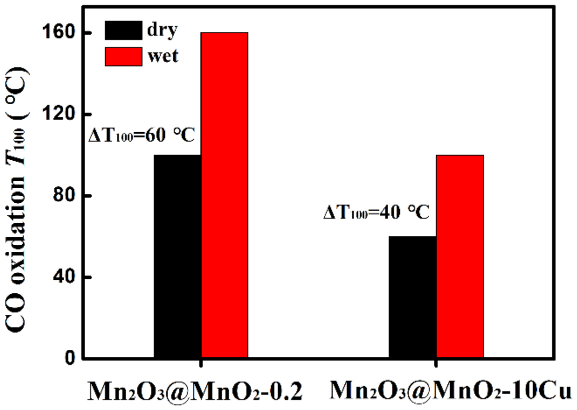

Figure S3. T_{100} of different catalysts under dry and wet gas conditions. Wet gas condition: the volume of water vapor in the feed gas is about 1.04 %.

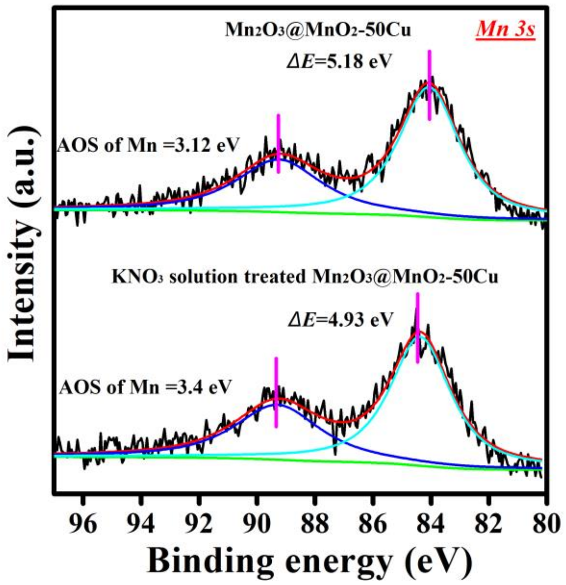

Figure S4. XPS spectra of Mn_2O_3@MnO_2-50Cu and KNO_3 solution treated Mn_2O_3@MnO_2-50Cu

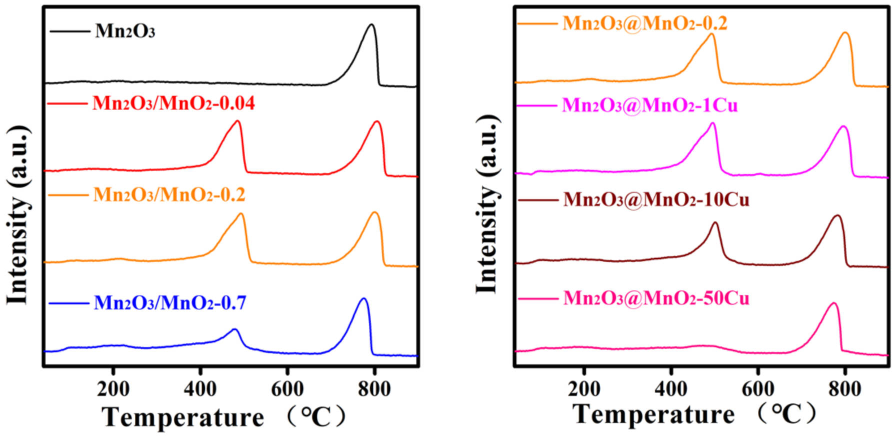

Figure S5. O_2-TPD profiles of different samples.

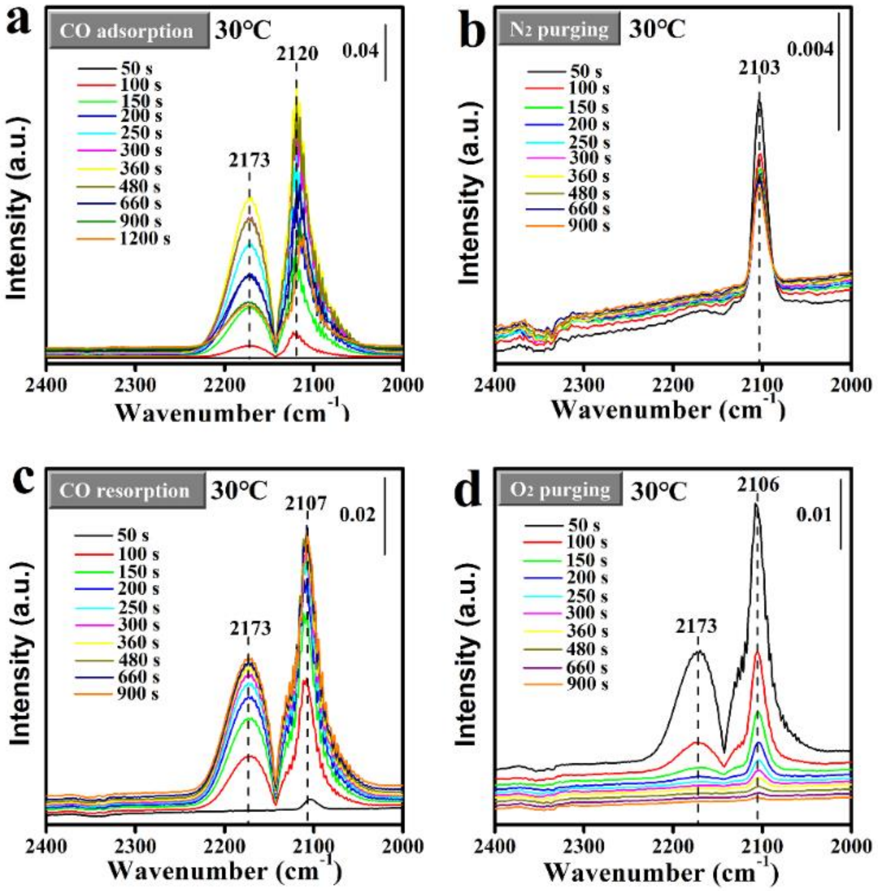

Figure S6. In situ DRIFTS study of (a) CO adsorption, (b) N_2 purging, (c) CO resorption, and (d) O_2 removal on Mn_2O_3@MnO_2-10Cu. The catalysts were pretreated in situ at 200 °C under mixture gas (50% O_2 and 50% N_2) flow in the DRIFTS reaction cell before data collection (5% CO flow rate, 10 mL·min^{-1}; N_2 flow rate, 20 mL·min^{-1}; O_2 flow rate, 20 mL·min^{-1}; temperature, 30 °C).

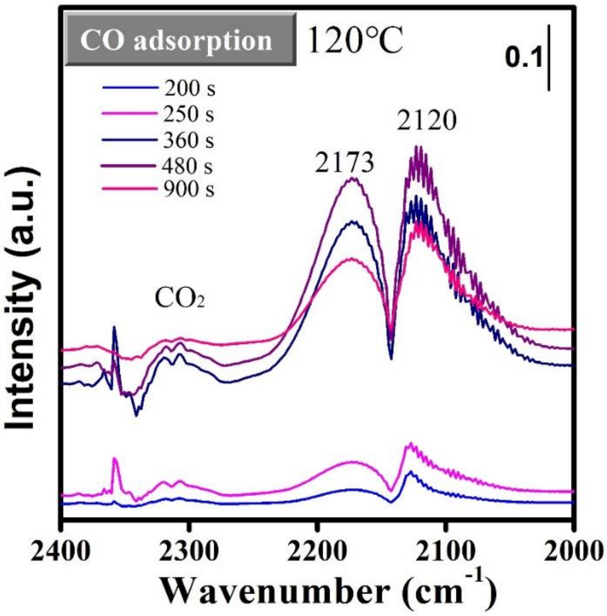

Figure S7. In situ DRIFTS study of CO adsorption on Mn_2O_3@MnO_2-10Cu. The catalysts were pretreated in situ at 200 °C under mixture gas (50% O_2 and 50% N_2) flow in the DRIFTS reaction cell before data collection (5% CO flow rate, 10 mL·min^{-1}; temperature, 120 °C).

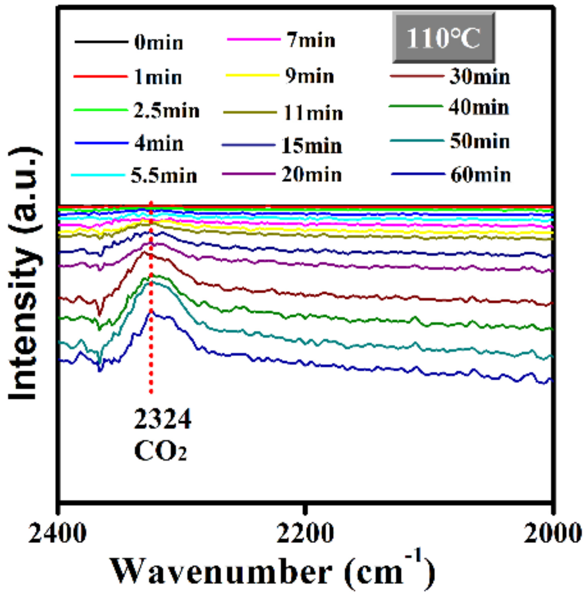

Figure S8. In situ DRIFTs spectra of Mn_2O_3@MnO_2-10Cu exposed to the flow of 120 ppm CO at 110 °C.

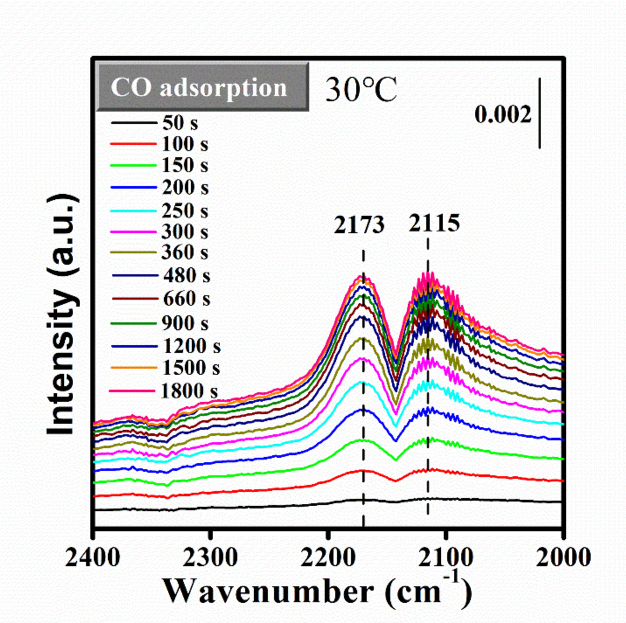

Figure S9. In situ DRIFTS study of CO adsorption on Mn_2O_3@MnO_2-10Cu under 2% CO wet gas (RH=100% at 25°C).
